# Empirical Evidence of a Bet‐Hedging Strategy in the Carrot Cyst Nematode *Heterodera carotae*

**DOI:** 10.1002/ece3.71918

**Published:** 2025-08-06

**Authors:** Sylvain Fournet, Didier Fouville, Catherine Porte, Josselin Montarry

**Affiliations:** ^1^ IGEPP, INRAE Institut Agro, Univ Rennes Le Rheu France

**Keywords:** bet‐hedging, biocontrol solution, fitness, hatching, *Heterodera carotae*

## Abstract

Bet‐hedging is an evolutionary strategy encountered in several plant and animal species that allows minimizing the risks by sacrificing mean fitness for a reduction in temporal fitness variation. The objective of the present work was to test the existence of a bet‐hedging strategy in plant‐parasitic cyst nematodes. The survival stage, the cyst, protects eggs containing second‐stage juveniles (J2) and can survive more than ten years in the soil. For some cyst nematode species, hatching of J2 mainly depends on a stimulation by root exudates of their host plant(s). The carrot cyst nematode *Heterodera carotae* is a good candidate for which a large proportion of juveniles hatch only in the presence of specific chemicals emanating from host roots. Hence, with this narrow host range and facing the impossibility of predicting the presence and quality of host plants, the hypothesis we tested here was that *H. carotae* should have developed a diversified temporal bet‐hedging strategy. Results from in vitro hatching tests demonstrated the existence of a bet‐hedging strategy: following a first stimulation by root exudates, the unhatched eggs that remained in the cyst were able to respond to a second stimulation. Moreover, the proportion of J2 hatching at the first or at the second stimulation was strongly impacted by the age of the cyst: older cysts responded better to a first stimulation than young ones. The fitness comparison of both batches of J2 suggested a small fitness advantage for early‐hatched juveniles, as they produced 20% more newly formed cysts than late‐hatched juveniles. Those results would have applied consequences for the development of a biocontrol strategy that stimulates the hatching of *H. carotae* juveniles in the absence of its host plant.

## Graphical abstract

Phytoparasitic cyst nematodes with narrow host range are expected to develop a diversified temporal bet‐hedging strategy as they cannot predict the presence and quality of host plants. The present work confirmed this hypothesis for the first time in a plant‐parasitic nematode, Heterodera carotae. Following a first stimulation by carrot root exudates, the unhatched eggs that remain in the cyst were able to respond to a second stimulation.

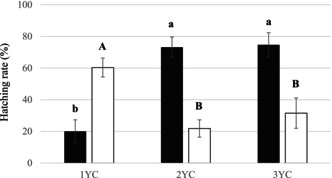

## 1 Introduction

In evolutionary theory, bet‐hedging is a successful adaptation that ensures long‐term population persistence for a given species in a fluctuating environment (Philippi and Seger 1989). In an unpredictable environment that exhibits no reliable cues for predicting future variation at the intergenerational scale, species may evolve a strategy without “knowing” how it will impact future fitness (Childs et al. 2010). In that case, fluctuating selection can favor the evolution of bet‐hedging (Kussel and Leibler 2005), which corresponds to sacrificing the mean fitness for a reduction of temporal fitness variation. It could be realized through a large panel of life history adaptations (Childs et al. 2010) and is observed from bacteria to humans (Venable 2007).

Bet‐hedging was originally proposed in annual plants for which a reservoir of ungerminated seeds was observed in the soil, i.e., a seed bank (Cohen 1966). This strategy, which is a diversified temporal bet‐hedging, allows minimizing risks for all the offspring to die in the case of adverse conditions if all the seeds germinate at the same time. Even so, the plant fitness is maximized when all seeds germinate; plants with seeds remaining in the seed bank will have a fitness advantage. Therefore, it can be advantageous for plants to produce some seeds that germinate the n‐year and some others that germinate the following year(s). Such a bet‐hedging through a seed bank was also highlighted in desert annual plants like *Lepidium lasiocarpum* (Philippi 1993) and in weeds (Roberts and Feast 1972). In desert annuals, Gremer and Venable (2014) found that germination rates were affected by the density of seeds in the seed bank. In weeds, germination rates decreased with soil depth (Roberts and Feast 1972). More broadly, it was shown that seed dormancy of bet‐hedging angiosperms is correlated to a higher polyphenol (flavonoid) content in seed coats (Renzi et al. 2020; Gianella et al. 2021).

Many invertebrate species are also known to use a bet‐hedging strategy to reduce the potential costs of a catastrophic event. *Diaptomus sanguineus*, an aquatic crustacean species, uses a form of diversified bet‐hedging called germ banking, in which emergence timing among offspring from a single clutch is highly variable (Evans and Dennehy 2005). Bet‐hedging through variable egg hatching patterns is seen in other crustaceans as well (Radzikowski 2013; Hakalahti et al. 2004). In nematodes, bet‐hedging strategies were described in *Nematodirus battus*, a nematode parasite of ruminants (van Dijk and Morgan 2010) and in the bacteriophage *Caenorhabditis elegans* (Chapman et al. 2024). In plant‐parasitic nematodes, and especially cyst nematodes, it was shown that larvae inside the cyst do not all hatch at the same time (see Perry and Gaur (1996) for a review), without using the term'bet‐hedgin'.

Every agricultural crop can be infested by at least one plant‐parasitic nematode species, and the most important plant‐parasitic nematodes are root‐knot nematodes (genus *Meloidogyne*) and cyst nematodes (genera *Heterodera* and *Globodera*) (Jones et al. 2013). The latter are particularly difficult to eradicate because they form cysts containing eggs that can persist for many years in soils. Cyst nematodes are sedentary endoparasitic pathogens that penetrate inside the plant roots as second‐stage juveniles (J2) and induce a feeding site, the syncytium, which is an important nutrient sink for the plant (Jones and Northcote 1972). They realize successive molts before becoming adult males and females. The males then leave the root to mate with females, which continue to feed and produce eggs until they die and form a cyst. These cysts contain hundreds of eggs, with one juvenile per egg (Perry et al. 2018).

With very low dispersal capabilities and often a very narrow host range, cyst nematodes have to face an unpredictable environment and have a large panoply of survival adaptations. For some species, their huge dependence on root exudates chemical cues to stimulate hatching (Perry 2002) partially resolves the 'risk' to hatch. Indeed, such a mechanism ensures that the emitting plant is a suitable host plant and that it is at a distance that allows its easy infestation by newly hatched juveniles. As an example, *Heterodera carotae* (Jones 1950) attacks only carrots, and a large proportion of juveniles hatch only after stimulation by root exudates from a suitable host plant, i.e., a plant belonging to the genus *Daucus*, with the exception of *Torilis leptophylla* (see Montarry et al. 2024 for a recent review). However, this ability to detect a suitable host gives no guarantee that it will survive long enough to allow the development of the penetrated juveniles. Indeed, once inside the roots, it takes around four to five additional weeks for juveniles to develop into mature adults and to produce fertilized females, which will form the cysts containing the next generation.

In addition to dependence on host cues, nematodes exhibit different types of dormancies that provide them with a range of strategies to synchronize hatching with unpredictable and seasonal environmental changes. Quiescence is a spontaneous and reversible response to unfavorable conditions released when favorable conditions return, while diapause is a stable arrest of development programmed in the nematode life cycle (Masler and Perry 2018). Moreover, it was shown for *H. sorghi* (Gaur et al. 1995) and for *H. glycines* (Masler et al. 2008) that cysts contain three different groups of eggs. The first group is fully hatching competent, and J2 hatch freely; the second group requires stimulation from host root exudates to hatch less or more quickly depending on the physiological stage of the eggs, and the last one, which does not hatch immediately, can be considered in diapause (Sommerville and Davey 2002; Masler and Rogers 2011; Masler et al. 2013). This last group ensures the survival of a part of the stock of J2 during an unfavorable period (including the intercrop in agriculture).

*Heterodera carotae* hatching rate was recently measured in vitro (Ngala et al. 2021; Gautier et al. 2021), under controlled conditions (Ngala et al. 2021) and in the field (Ngala et al. 2024). Those different studies also showed that hatching stimulation by root exudates never reached 100%, indicating that some viable juveniles stay in the cyst and thus raised the question of their ability to hatch later. The aim of the present study was thus to test the existence of a bet‐hedging strategy in the carrot cyst nematode *H. carotae*. The hypothesis was that *H. carotae* uses a diversified temporal bet‐hedging strategy with eggs that hatch at a first stimulation by root exudates and a bank of unhatched eggs that will hatch at a second stimulation. We have also explored here if this strategy depends on the age of cysts and if it confers a fitness advantage to one or the other groups, i.e., early and late‐hatched juveniles.

## 2 Methods and Materials

### 2.1 Two Successive Hatching Stimulations

Three distinct cohorts of *H. carotae* cysts were sampled the same day in autumn 2021 in three distinct fields (located on the west coast of Normandy, France, near Créances) where carrot was produced in the current year (2021), one year ago (2020), and two years ago (2019). A Kort elutriator was used to extract the cysts, which were thus one‐year cysts (1YC), two‐year cysts (2YC), and three‐year cysts (3YC).

Root exudate of the carrot cultivar Touchon was produced by soil leaching as described in Ngala et al. (2021). Carrots were cultivated from seeds to a 7‐week growth stage in 1 L pots filled with 2/3 sand/land soil under glasshouse regulated at 21°C/17°C day/night temperatures, with a photoperiod of 16 h. Fifteen pots, with five to seven plants per pot, were left to stand for 24 h without application of water before being saturated by slowly pouring osmotic water from the top of the pot. Following pot saturation, the pots were each suspended upon a beaker before 200 mL of osmotic water was added from the top of the pot and allowed to leach into the beaker underneath the pot. Root exudates collected from the different pots were pooled, filtered through cellulose Watman filters, and stored at −20°C until required for the experiment.

To perform a first hatching stimulation, twelve cysts of each cohort were individually placed in sieves that were put in a 12‐well plate containing carrot root exudate. Sieves allowed the passage of hatched juveniles but not of cysts. The number of hatched juveniles was counted under a stereomicroscope every five days for 30 days, and the exudate was renewed at each counting.

Each cyst was then individually stored in humid sand at 4°C for four months. A second hatching stimulation was performed following the same protocol and the same exudate. At the end of the second stimulation, cysts were crushed and the number of remaining viable juveniles was counted under a stereomicroscope. The hatching rates of both successive stimulations and the cumulated hatching rate were then calculated.

### 2.2 Fitness Comparison Between Early and Late‐Hatched Juveniles

Seven thousand and fifty cysts from the cohort of 1YC sampled in 2022 were used for this experiment one year later (and thus became two‐year cysts) to collect juveniles hatched after a first stimulation (early) and juveniles hatched after a second stimulation (late). To perform this synchronization, 600 cysts (i.e., 4/5) were stimulated the first time during 30 days in root exudate, which was renewed every five days, and then placed three months at 4°C. The remaining 150 cysts (i.e., 1/5) were directly placed at 4°C, i.e., for four months. All cysts were then simultaneously stimulated for hatching to obtain the two batches of juveniles: Early and Late.

The early batch was inoculated into 62 plants of the carrot cultivar Touchon with 200 J2 per plant, and the late batch was inoculated into 63 plants with 200 J2 per plant. The plants were individually cultivated in 70 mL small pots filled with a 4/1 sand/kaolin mixture, which were randomized in a climatic chamber regulated at 20°C/18°C day/night temperatures, with a photoperiod of 16 h. J2 were inoculated at the 2‐leaves stage. Newly formed cysts were extracted from the sand/kaolin mixture after 60 days. They were counted and then placed in humid sand at 4°C for six months. The size of newly formed cysts was measured using 10 cysts randomly selected from 30 replicates, i.e., 30 plants randomly selected in each batch (early and late). The size of each cyst was evaluated through the surface area using a magnifying stereomicroscope coupled with image analysis software (Microvision Instruments, Histolab v8.1.0, Evry, France). The hatching dynamics were recorded for each replicate every 4–5 days during 32 days using the carrot root exudate Touchon. At the end of the hatching test, cysts were crushed to count the remaining viable juveniles. This allowed for the calculation of the hatching rate but also the number of juveniles per cyst.

### 2.3 Statistical Analysis

All statistical analyses were performed using the R software version 4.1.2 (R Core Team, 2021). Normality and homogeneity of variances were checked using theShapiro‐Wilk and the Levene tests, respectively.

For each hatching stimulation (i.e., the first and the second stimulation), the age effect was tested for the hatching rate using a one‐way ANOVA. The batch effect (Early vs. Late) was tested for the number of cysts, the size of cysts, the hatching rate, and the number of juveniles per cyst using a one‐way ANOVA. Multiple comparisons of means were performed using the Tukey test (*α* = 0.05).

## 3 Results

### 3.1 A Bet‐Hedging Strategy and a Strong Effect of the Age of the Cysts

**FIGURE 1:**
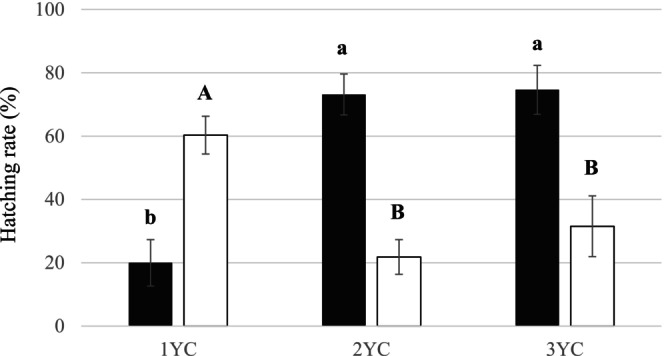
Hatching rate (mean ± SEM) of three cohort of cysts, i.e., one‐year cysts (1YC), two‐years cysts (2YC) and three‐years cysts (3YC), after a first stimulation by carrot root exudates (black histograms) and after a second stimulation (white histograms). Letters represent the homogenous groups identified by the Tukey test at the 5% threshold.

After a first hatching stimulation by carrot root exudates and four months at 4°C, juveniles remaining in the cysts were clearly able to respond to a second hatching stimulation (Figure 1). The hatching capability of the unhatched juveniles after the first stimulation was not lost.

At the end of the first hatching stimulation, the hatching rate was highly variable, with a strong effect of the age of the cysts (*F*_2,33_ = 18.77; *p* < 0.0001). While the hatching rate of two‐year cysts and three‐year cysts was high (73% and 75%, respectively), the hatching rate of 1YC was strongly reduced; only 20% of the juveniles hatched (Figure 1).

The age effect was also significant at the end of the second hatching stimulation (*F*_2,33_ = 10.99; *p* = 0.0002), with a higher hatching rate for the 1YC (60%) than for the two‐year cysts and three‐year cysts (22% and 32%, respectively) (Figure 1).

Consequently, after two hatching stimulations, the cumulative hatching rate was very high for the three cohorts of cysts, from 80% to 99% (Table 1). The statistical analysis showed that J2 within two‐year cysts and three‐year cysts have hatched more than J2 within 1YC (*F*_2,33_ = 8.83; *p* = 0.0009).

**TABLE 1:** Mean hatching rates of the three cohorts of cysts (one‐year, two‐years and three‐years cysts) after the first (stimulation 1) and the second (stimulation 2) stimulation by carrot root exudates. Means of the cumulative hatching rate and the corresponding standard error of mean (sem) are indicated and statistically compared. Letters represent the homogenous groups identified by the Tukey test at the 5% threshold.

Cohort	Stimulation 1	Stimulation 2	Cumulative hatching rate	sem	Statistical group
1YC	19.98	60.32	80.31	5.42	B
2YC	73.16	21.84	95.00	1.83	A
3YC	74.63	55.92	99.01	0.45	A

### 3.2 A Small Fitness Cost Associated With the Bet‐Hedging Strategy

Life history traits were compared between nematodes hatched during the first stimulation and nematodes hatched during a second stimulation by root exudates.

**FIGURE 2:**
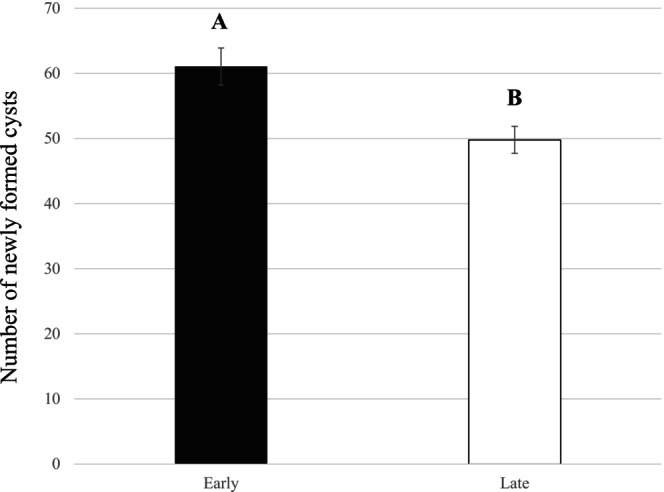
Number of newly formed cysts on carrots cv. Touchon inoculated by early‐hatched juveniles (early) and late‐hatched juveniles (late). Letters represent the homogenous groups identified by the Tukey test at the 5% threshold.

A cycle of multiplication on the carrot cultivar Touchon showed that early‐hatched juveniles produced more newly formed cysts than late‐hatched juveniles (*F*_1,123_ = 10.25; *p* = 0.0017). The inoculation of 200 J2 led to a mean of 61 cysts per plant for early‐hatched compared to a mean of 50 cysts per plant for the late‐hatched juveniles (Figure 2).

**FIGURE 3:**
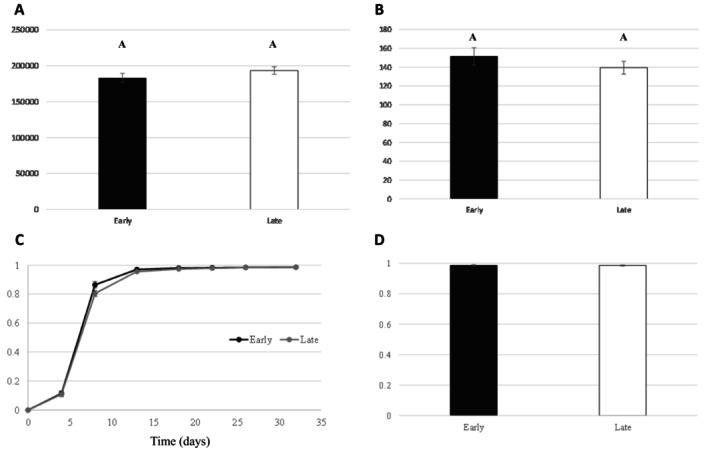
Size (in μm^2^) of newly formed cysts on carrots cv. Touchon inoculated by early‐hatched juveniles (early) and late‐hatched juveniles (late) (A). Number of juveniles per cyst in newly formed cysts (B). Hatching capability of juveniles contained in newly formed cysts: Hatching dynamics over time (C) and hatching rate after 32 days in carrot root exudates (D). Letters represent the homogenous groups identified by the Tukey test at the 5% threshold.

The size of newly formed cysts was measured, using 10 cysts from 30 plants in each batch, and results showed no significant difference between cysts produced from early‐hatched and from late‐hatched juveniles (*F*_1,58_ = 1.60; *p* = 0.212; Figure 3A). Those newly formed cysts contained a mean of 151 J2 per cyst for the ones produced from early‐hatched juveniles, and 139 for the ones produced from late‐hatched juveniles, with no significant difference (*F*_1,58_ = 1.083; *p* = 0.302; Figure 3B). There is also no difference in the hatching dynamic (Figure 3C) and in the final hatching rate which reaches 98.6% for each batch of cysts (*F*_1,58_ = 0.002; *p* = 0.964; Figure 3D).

## 4 Discussion

Using natural field populations of the carrot cyst nematode *H. carotae* and an in vitro test to stimulate two successive times the hatching of the juveniles by carrot root exudates, our results clearly demonstrated the existence of a bet‐hedging strategy in this plant‐parasitic nematode. Following a first stimulation, the unhatched eggs that remained inside the cyst were able to respond to a second stimulation. The fitness comparison of both batches of juveniles (early‐ and late‐hatched juveniles) suggested a small fitness advantage for early‐hatched juveniles: they produced 20% more newly formed cysts than late‐hatched juveniles, but those newly formed cysts were identical in terms of size, eggs' content, and hatching capability. Moreover, the proportion of juveniles hatching at the first or at the second stimulation was strongly impacted by the age of the cyst: old cysts (more than one year) responded better to a first stimulation than young ones (1YC).

In the evolutionary history of *H. carotae*, the hatching trait, which could be considered as the first step of its life cycle, appears to have been strongly optimized. First, the nematode was selected to be able to detect one (or more) molecule(s) specific to root exudates of carrots, which remain unidentified but are likely essential for the plant as it has not been counter‐selected during the coevolutionary process between the plant and the nematode. This confers a huge advantage to the nematode as it could stay for years in its survival state, the eggs within the cyst, in the absence of the host. Second, Greco et al. (1982) showed that the age of carrot plants affected the hatch of *H. carotae* and that most juveniles emerged with root exudates from 5 to 7‐week‐old carrots. This allows the nematode to attack roots of the optimal stage: if it penetrates too young roots, it would kill the host, and if it waits too long, the roots would be more difficult to penetrate. This adaptive ability confers a second advantage to the nematode. Moreover, it seems that a bet‐hedging strategy was also selected in *H. carotae*, with juveniles that do not hatch during the first stimulation. So, the hatching trait has been selected to recognize the host at the best phenological stage, but the temporal diversification of this trait has also been a target for natural selection.

We showed that bet‐hedging can take two different forms according to the tested field populations. It was assumed here that it was an effect of the age of cysts because a previous population genetics study showed that *H. carotae* populations from this carrot production area were genetically similar (Esquibet et al. 2020), but since there was only one population per cohort of cysts (no replicate), we cannot exclude that these populations were different due to other unidentified attributes. However, this age effect could be a consequence of differences in hatching abilities for the offspring present in the cyst according to their age. If we assume that *H. carotae* hatching behavior is close to the one described by Masler et al. (2008) for *H. glycines* , eggs in newly formed cysts of *H. carotae* should be divided into three hatching groups: the first one constituted of eggs fully hatching competent, the second one constituted of a continuum of eggs at different physiological development stages that will hatch more or less quickly if stimulated by host cues, and the third one constituted of eggs that can be assumed to be in diapause, which cannot hatch. As a result, the low hatching rate we observed for 1YC at the first stimulation would correspond to the hatching of the eggs of the first group and to a part of the second. The storage of these cysts for three months at 4°C would have allowed the unhatched eggs belonging to the second and third groups to reach a physiological stage compatible with hatching and to come out of diapause, respectively. Moreover, we can hypothesize that the relative proportion of each group should evolve according to a trade‐off between hatching and staying more time in the cyst. The more time passes, the less advantageous it is to stay in the cyst, with an increase in the risk for the larvae of being predated, parasitized, or dying consecutively to abiotic factors (high temperature, for instance) or mechanical action. Indeed, the proportion of hatching larvae after a first stimulation could increase with the age of the cyst until representing the highest proportion. This second hypothesis could be tested with older cysts than those we used.

Limiting the number of hatched juveniles may also be a way for the nematode to limit competition. Indeed, in plant‐parasitic nematodes, competition occurs at two distinct steps. The first one is due to the limited number of root sites of penetration, a finite quantity, which leads to the death of unpenetrated juveniles. The second one occurs inside the root: the higher the number of juveniles in a root, the higher will be the proportion of males. Consequently, the competition intensity is negatively correlated to the multiplication rate (Mugniéry and Bossis 1985). Moreover, bet‐hedging could also theoretically reduce mating between individuals coming from the same cyst, i.e., consanguineous mating. Consanguinity is possibly reduced but not enough to reach the Hardy–Weinberg equilibrium, as positive *F*_*IS*_ were reported in *H. carotae* populations (Gautier et al. 2019; Esquibet et al. 2020).

While the hatching has been a widely studied trait for a long time (e.g., Shepherd and Cox 1967), it was primarily considered as a biological feature of cyst nematodes and not as a selected evolutionary strategy even if Masler and Perry (2018) point out that hatching responses of cyst nematodes are clearly linked to their capability to survive. An outstanding question now is whether what we have shown for *H. carotae* could be true for several nematode species with distinct levels of host specialization and distinct levels of consanguineous mating. The potato cyst nematodes, *Globodera pallida* and *G. rostochiensis*, also have a narrow host range, as they attack only Solanaceous plants, while *Heterodera schachtii* is able to attack a large range of hosts from different plant families. The comparison between *G. pallida* and *G. rostochiensis* could also be interesting as populations of the former are characterized by a strong heterozygote deficit (*F*_*IS*_ > 0; e.g., Montarry et al. 2015) while populations of the latter are at the Hardy–Weinberg equilibrium (*F*_*IS*_ = 0; e.g., Esquibet et al. 2024). For all those cyst nematode species, females are mated by several males (Green et al. 1970; Triantaphyllou and Esbenshade 1990), and polyandry could also be seen as a bet‐hedging strategy because it allows females to minimize risk associated with uncertainty around male suitability. Such a bet‐hedging via polyandry was demonstrated in the red flour beetle *Tribolium castaneum* (Matsumura et al. 2021).

The extent to which bet‐hedging represents an optimal strategy is predicted to depend on the level of environmental variation, with highly stochastic environments favoring a high degree of bet‐hedging (Fenton and Hudson 2002). The bet‐hedging strategy in *H. carotae* must have been selected in the wild, where the level of environmental variation is high and plants die frequently. In more predictable environments, bet‐hedging has a cost since a proportion of offspring will be unable to take advantage of favorable conditions for population growth. So, this strategy could be under counter‐selection in cultivated fields, where plants are pampered by producers and may offer an optimal food source that optimizes the nematode fitness. An interesting prospect would be to study the occurrence of *H. carotae* on wild Daucus species and to quantify and compare their hatching behavior with populations sampled on cultivated carrots.

In the end, the fitness comparison between early‐ and late‐hatched juveniles suggested a fitness advantage for early‐hatched juveniles, as they produced 20% more newly formed cysts than late‐hatched juveniles. However, this comparison was performed under optimal conditions while the late‐hatched juveniles might have an advantageous unidentified feature under stressful environments, as was the case for some bacteria for which slow‐growing cells survive antibiotic exposure (Balaban et al. 2004). For the other measured traits (size of cysts, number of juveniles per cyst, and hatching capability), there was no significant difference. One might have expected, if the characteristic is genetically determined, that late‐hatched juveniles could produce eggs that hatch later or with more difficulties, but this is absolutely not the case. There is currently no explanation of how the bet‐hedging functionally works in *H. carotae*, and further studies are needed to explore potential physical differences between eggs able and not able to respond to a first stimulation by root exudates. A transcriptomic approach would also be performed using early‐ and late‐hatched juveniles to identify differently expressed genes.

Plant‐parasitic nematodes cause considerable economic losses in agriculture: the worldwide crop losses due to plant‐parasitic nematodes have been estimated at around $173 billion per year (Elling 2013). From an applied point of view, our results would have practical consequences. The strong dependence of *H. carotae* on carrot root exudates is indeed a feature that could allow the use of exudates as a new biocontrol product that stimulates the hatching of *H. carotae* juveniles in the absence of its host plant. The efficacy of this ‘lure and starve’ or ‘suicide hatching’ strategy was demonstrated under control conditions (Ngala et al. 2021) and then in field trials (Ngala et al. 2024). Moreover, and contrary to resistant plant cultivars or chemical nematicidal products, which may respectively select for virulent and resistant individuals, the use of root exudates would select for individuals that are unable to hatch. Unfortunately, the bet‐hedging strategy highlighted here showed that the unhatched juveniles that stay in the cyst have preserved their ability to hatch later. Finally, our result about the age of cysts would allow to optimize the positioning of this control method in the rotation plan, in order to apply exudates on cysts which are more than one‐year old, i.e., which will be highly receptive to a stimulation with root exudate.

## Author Contributions

**Sylvain Fournet:** conceptualization (equal), formal analysis (equal), methodology (equal), supervision (equal), writing – original draft (equal), writing – review and editing (equal). **Didier Fouville:** investigation (equal), methodology (equal), writing – review and editing (equal). **Catherine Porte:** investigation (equal), methodology (equal), writing – review and editing (equal). **Josselin Montarry:** conceptualization (equal), formal analysis (equal), funding acquisition (lead), methodology (equal), supervision (equal), writing – original draft (equal), writing – review and editing (equal).

## Conflicts of Interest

The authors declare no conflicts of interest.

## Data Availability

All data used in this article are available at data.inrae.fr (https://doi.org/10.57745/6VK0YD).
